# Analysis of the cost composition and influencing factors of Chinese medicine advantageous diseases

**DOI:** 10.1097/MD.0000000000044447

**Published:** 2025-09-26

**Authors:** Chenhao Ma, Xiping Li, Tong Qin, Songkun Ding, Wei Hu, Tao He

**Affiliations:** a School of Management, Hubei University of Chinese Medicine, Wuhan, Hubei, China.

**Keywords:** cost composition, Gray relational analysis, health insurance payment reform, length of stay, TCM-privileged diseases

## Abstract

With rising medical costs and increasing health insurance payment pressures in China, optimizing resource allocation and enhancing diagnosis efficiency for TCM (traditional Chinese medicine)-privileged diseases (e.g., stroke, facial paralysis, bone bi-syndrome) has become critical. These diseases, valued for their standardized protocols and curative advantages, are prioritized in national policies. This study analyzes their cost composition and core drivers to inform medical cost control and policy optimization. Using 2023 outpatient/inpatient data from pilot hospitals in Hubei (782 stroke, 294 facial paralysis, 244 bone bi-syndrome cases), Gray relational analysis and multiple linear regression quantified cost structures and influencing factors. Data were logically reviewed, excluding cases with abnormal hospitalization days (<1 or >180 days) or unreasonable costs, focusing on TCM treatment fees, diagnostic costs, and length of stay (LOS). TCM treatment fees dominated costs (41% for stroke, 48.05% for facial paralysis, 33.4% for bone bi-syndrome), driven by diverse therapies like acupuncture and herbal medicine. Diagnostic fees followed (16.2%, 15.25%, 22.14%), linked to imaging/laboratory tests. LOS was the strongest driver of total hospitalization costs (standardized coefficients: 0.746 for stroke, 0.697 for facial paralysis, 0.548 for bone bi-syndrome). Age and gender showed partial significance in univariate analysis but not in multivariate models. Cost structures are TCM-treatment-oriented, with LOS as the key cost driver. Recommendations include optimizing health insurance payment weights via diagnosis-related group-differentiated compensation, refining hospitalization management to reduce unnecessary LOS, and building multidimensional cost-effectiveness research networks integrating machine learning and cost-effectiveness analysis to balance costs and efficacy for sustainable TCM development.

## 1. Introduction

In recent years, China has made significant progress toward a moderately prosperous society, raising public expectations for a higher quality of life. As the national healthcare system improves, healthcare costs have concurrently risen. According to the China Health Statistics Yearbook, per capita healthcare spending increased from 463.90 dollars in 2016 to 837.61 dollars in 2022, while the share of healthcare costs relative to gross domestic product grew from 6.21% to 7% during the same period, placing substantial pressure on government finances.^[1]^ Moreover, irrational healthcare spending remains a global issue that undermines the efficiency of health investments.

As an important concept of Chinese medicine, the dominant disease types of Chinese medicine can better define the dominant areas of Chinese medicine, strengthen the standardization of Chinese medicine in the treatment of diseases, and make greater contributions to safeguarding people’s health and building a healthy China. In the 2024 government work report, special emphasis was placed on “upgrading the capacity of healthcare services, promoting the synergistic development and governance of healthcare insurance, healthcare and medicine, strengthening the normative supervision of healthcare insurance funds, promoting the inheritance and innovation of traditional Chinese medicine (TCM), as well as strengthening the construction of TCM advantageous specialties.”^[2]^

This study uses data from pilot hospitals in Hubei Province that implement a collection and payment system for Chinese medicine advantageous diseases. We analyze both outpatient and inpatient cost compositions and the factors influencing these costs to assess the economic burden borne by patients. Based on our findings, we propose targeted cost control measures to reduce patients’ financial burdens.

## 2. Literature review

The composition of in-hospital expenses for TCM-dominant diseases is characterized by a high proportion of pharmaceuticals and examinations, coupled with a low proportion of technical services. Notably, the structure has undergone significant changes in recent years. Multiple studies have revealed this trend through structural variation analysis. For instance, a study on distal radius fracture cases at Sichuan Orthopedic Hospital by Zhang et al^[3]^ found that between 2016 and 2020, examination fees, TCM treatment fees, and pharmaceutical costs accounted for a large proportion with positive changes (high contribution rates). In contrast, medical service fees, nursing fees, and acupuncture fees, which reflect the service value of medical staff, showed negative changes. Delving into specific disease categories, Xu et al^[4]^ based on an analysis of 190,000 in-hospital cases in Beijing, found that the use of Chinese patent medicines significantly increased costs in the treatment of TCM-dominant diseases (compared to nonuse of Chinese medicines). However, Chinese herbal decoctions reduced costs in secondary hospitals while increasing them in tertiary hospitals, indicating institutional differences in the relationship between TCM intervention models and costs. An earlier comparative study by Ji^[5]^ similarly suggested that patients using Chinese herbal decoctions had significantly higher total hospitalization costs, pharmaceutical expenses, and average daily hospitalization costs than nonusers, revealing cost differences in TCM treatments, albeit without fully controlling for variations in disease complexity.

The reform of DRGs (diagnosis-related groups) payment has initially shown effectiveness in controlling costs for TCM-dominant diseases, but it has also highlighted challenges in adapting the payment mechanism to the characteristics of traditional TCM diagnosis and treatment. A comparison of data on patients with ischemic stroke (sample size: 9421 cases) before and after DRG implementation by Chen et al^[6]^ indicated that after the reform, the average hospitalization expense per case, pharmaceutical costs, and treatment fees decreased significantly. The cost structure was dominated by pharmaceutical fees (44.95%) and diagnostic fees (39.45%), with regression analysis confirming a negative correlation between DRG payment and costs. From the perspective of stakeholders, Peng et al^[7]^ pointed out that medical insurance payment reforms alter the behavior of service providers through economic incentives, but their policy design must balance the demands of multiple parties. However, the current DRG framework inadequately reflects TCM characteristics. Gu^[8]^ reflected that the DRG reform exhibits tendencies toward “scientism, elitism, and mysticism,” with administrative dominance and a lack of negotiation mechanisms with service providers, particularly failing to account for the flexibility of TCM’s syndrome differentiation and treatment. Based on cases from TCM hospitals, Zhang et al^[3]^ proposed that payment policies should be synchronously adjusted following increases in TCM service prices; otherwise, the undervaluation of service value cannot be fundamentally addressed. Zhang et al^[9]^ further revealed the common limitation of payment methods prioritizing “cost control” over “quality incentives,” which may inhibit the application of characteristic TCM technologies in the TCM field.

Hospital type, level, and management strategies significantly influence the cost level and structure of TCM-dominant diseases, with cost differences primarily stemming from resource input intensity and diagnostic/treatment models. A study by Liu et al^[10]^ analyzing 95 diseases across 47 hospitals found that total costs in general hospitals were higher than those in TCM hospitals but lower than in integrated TCM-Western medicine hospitals. The average daily cost in general hospitals was significantly higher than in TCM-type hospitals, while the length of stay (LOS) was relatively shorter, reflecting differences in efficiency pathways among various institutions. A regression model by Xu et al^[4]^ confirmed that hospital level, type, and LOS are common factors affecting the utilization rate of TCM. The higher costs of Chinese herbal decoctions in tertiary hospitals may be associated with the concentration of complex cases and the use of high-cost equipment. A study on single-disease management by Tao^[11]^ indicated that controlling ineffective hospitalization days is a key measure to reduce costs. Similarly, a multiple regression analysis by Yan et al^[12]^ identified the LOS as the primary factor influencing costs. Notably, the “public contract model” proposed by Gu^[8]^ emphasizes that payment reforms should incentivize hospitals to provide cost-effective services, but a performance evaluation system specific to TCM must be established.

Existing studies have systematically revealed the characteristics and dynamic changes in the cost composition of TCM-dominant diseases: pharmaceutical and examination fees contribute significantly, while the value of TCM characteristic technical services has long been undervalued. Cost differences among institutions mainly result from service intensity and resource input models. The DRG payment reform has initially achieved cost control goals but suffers from issues such as prioritizing Western medicine over TCM and rigid mechanisms, failing to fully address the individualized needs of TCM’s syndrome-based treatment. Research gaps are concentrated in 3 areas: first, the attribution analysis of cost structure changes lacks specificity to TCM diseases, with unclear structural contributions of internal components of TCM treatment fees (decoctions/patent medicines/non-pharmaceutical therapies); second, existing studies on cost-influencing factors mostly focus on conventional variables (e.g., LOS, institution type) and insufficiently quantify the cost effects of key TCM diagnostic and treatment elements (e.g., syndrome differentiation types, treatment principles, combinations of TCM characteristic technologies); third, research on TCM payment reform pathways remains in the theoretical discussion stage, lacking TCM value quantification standards and pilot data verification adapted to DRG/Diagnosis-Intervention Packet.

Based on data from outpatient and inpatient cases in 2023 from pilot hospitals in Hubei Province, this study covers 3 TCM-dominant diseases: stroke, facial paralysis, and bone bi (bone impediment). It combines Gray relational analysis (GRA) with a multiple linear regression model to overcome the one-sidedness of traditional single-factor analysis. When analyzing the cost composition of stroke, facial paralysis, and bone bi, GRA is used to quantify the correlation between each cost item and total costs, enabling precise identification of core driving factors. Meanwhile, the multiple regression model controls for confounding variables such as age and gender.

## 3. Materials and methods

### 3.1. Source

We selected the first-page records of inpatient and outpatient cases from a public hospital in Hubei Province (discharge dates: January 1, 2023 to January 1, 2024) that participated in the pay-per-disease policy for Chinese medicine advantageous diseases.

Data processing involved verifying patients’ dates of birth to validate age, reviewing profile information (e.g., specific medical conditions) to confirm gender, and excluding records with logical errors (such as hospitalization durations of <1 day or more than 180 days and costs below 13.94 dollars).

#### 3.1.1. Medical cost components

In this thesis, “medical service” refers to both drug services and diagnosis and treatment services. The clinical pathway cost analysis is based on first-page records from a Chinese medicine hospital. The total hospitalization cost is categorized as follows:

1. Comprehensive medical service fee: includes general medical service fee, general treatment operation fee, nursing fee, and other related fees.
2. Diagnostic fee: encompasses pathological diagnosis fee, laboratory diagnosis fee, diagnostic imaging fee, and clinical diagnosis item fee.
3. Treatment fee: covers both nonsurgical and surgical treatment fees.
4. Rehabilitation fee.
5. Chinese medicine service fee: consists of the Chinese medicine diagnostic fee, syndrome differentiation diet therapy (SDDT), and other Chinese medical service fees (which include Chinese medicine diagnosis, dietary treatment, special preparation and processing of Chinese medicine, and Chinese medicine treatment [such as Chinese orthopedics and traumatology, acupuncture and moxibustion, tui-na, treatment of Chinese anal and intestinal conditions, and other specialized Chinese medicine therapies]).
6. Western medicine fee.
7. Chinese medicine fee: involves fees for proprietary Chinese medicines and Chinese herbs.
8. Blood and blood products fee.
9. Consumable material fee: covers disposable medical materials used for examinations, treatments, and surgical procedures.
10. Other fees.

#### 3.1.2. Diagnostic criteria

All medical records with a discharge principal diagnosis matching the following disease names and complete charge information were included in the analysis:

1. Stroke disease (acute phase of ischemic stroke), TCM diagnosis: first diagnosis of stroke disease (TCD: BNG080) Western medicine diagnosis: first diagnosis of stroke (ICD-10: I63).
2. Facial paralysis disease (facial neuritis), Chinese medicine diagnosis: first diagnosis of facial paralysis disease (TCD: BNV120) Western medicine diagnosis: first diagnosis of facial neuritis (ICD-10: G51.802).
3. Bone paralysis (osteoarthritis), TCM diagnosis: first diagnosis of Inibs (TCD: BNO100) Western medicine diagnosis: first diagnosis of rheumatoid arthritis (ICD-10:M06.9).

### 3.2. Research methodology

We analyzed outpatient and inpatient medical costs to identify the primary factors influencing the expenses associated with Chinese medicine advantageous diseases and to explore effective methods for reducing the economic burden on patients and society. To examine the cost accumulation characteristics of TCM treatments, which involve multiple steps over extended periods, we employed gray correlation analysis to quantify the strength of correlation for each cost category and supplemented this with a multiple linear regression model to control for confounding factors. In this analysis, gender, patient age, and the number of hospitalization days were used as independent variables to overcome the limitations of traditional univariate analysis.^[13]^ Disease category data were integrated and entered into SPSS for analysis using a combination of descriptive statistics, mean tests, logistic regression, correlation tests, 1-way ANOVA, gray correlation analysis, and multiple linear regression. Descriptive statistics provided an overview of general data, while multiple linear regression was used to determine standardized costs for each disease category. Statistical significance was assessed using a threshold of *P* < .05.

The GRA is a comprehensive evaluation method for studying the correlation between sequences. It measures the strength of correlation by calculating the relational degree between characteristic sequences and the parent sequence.

Single-factor analysis is used to compare the mean differences among 3 or more independent groups, and it is applicable to continuous dependent variables and categorical independent variables.

Multiple linear regression analysis is employed to explore the influence of multiple independent variables (*X*) on a single continuous dependent variable (*Y*). It serves as a core method in empirical research to verify causal hypotheses between variables.

## 4. Analysis of factors influencing the cost of hospitalization for “stroke”

### 4.1. Cost composition of disease types

#### 4.1.1. Related to hospitalization costs

From January 1, 2023 to December 31, 2023, a total of 1 year, 782 stroke patients were discharged from the sample hospital. The average total hospitalization cost for the 782 patients was 2343.15 dollars, with the maximum value of the cost being 6656.19 dollars and the minimum value being 290.70 dollars. Among them, the average cost of general medical service was 101.83 dollars, the minimum value was 14.22 dollars, the maximum value was 508.98 dollars, and the average percentage of general medical service was 4.35%; the average cost of nursing care was 27.23 dollars, the minimum value was 2.79 dollars, and the maximum value was 118.74 dollars, and the average percentage of nursing care was 1.16%. The average cost of diagnosis was 379.86 dollars, the minimum value was 14.63 dollars, the maximum value was 2065.03 dollars, and the average proportion of diagnosis cost was 16.2%; the average cost of TCM treatment was 961.06 dollars, the minimum value was 0 dollars, the maximum value was 3664.85 dollars, and the average proportion of TCM treatment cost was 41%; the average cost of Western medicine was 303.37 dollars, the minimum value was 0 dollars, the maximum value was 5491.95 dollars, and the average proportion of Western medicine cost was 12.95%; the average cost of proprietary Chinese medicine was 99.22 dollars, the minimum value is 0 dollars, the maximum value is 668.30 dollars, and the cost of proprietary Chinese medicines accounted for an average of 4.23%; the cost of Chinese herbal medicines was 80.95 dollars on average, the minimum value is 0 dollars, the maximum value is 783.35 dollars, and the cost of Chinese herbal medicines accounted for an average of 3.45%; the cost of materials was 49.33 dollars on average, the minimum value is 0.56 dollars, the maximum value is 641.50 dollars, and the cost of materials accounted for an average of 2.95%; the cost of Western medicines accounted for an average of 12.95%. The average cost of materials was 49.33 dollars, the minimum value was 0.56 dollars, the maximum value was 641.50 dollars, and the average cost of materials was 2.1%. The components of wind hospitalization costs are shown in Table 1.

**Table 1:** Analysis of stroke hospitalization cost components (unit: dollars).

Sports event	Minimum value	Average value	Maximum values
Total cost of hospitalization	290.70	2343.15	6656.19
General medical services	14.22	101.83	508.98
Nursing costs	2.79	27.23	118.74
Diagnostic fee	14.63	379.86	2065.03
TCM treatment fee	0	961.06	3664.85
Western drug costs	0	303.37	5491.95
Cost of proprietary Chinese medicines	0	99.22	668.30
Chinese herbal medicine costs	0	80.95	783.35
Material cost	0.56	49.33	641.50

Source: Primary data collection.

TCM = traditional Chinese medicine.

#### 4.1.2. Gray correlation analysis of hospitalization costs

Using the average total hospitalization cost as the reference, we calculated the absolute differences between each individual cost component for stroke and the average total cost. Based on the maximum and minimum differences, correlation coefficients were computed for each cost component. Table 2 summarizes these results. In descending order, the correlation coefficients with total hospitalization cost are as follows: TCM treatment fee, diagnostic fee, Western drug costs, general medical services, cost of proprietary Chinese medicines, Chinese herbal medicine costs, material cost, and nursing costs. Notably, the ranking from the gray correlation analysis mirrors that obtained from the percentage breakdown of hospitalization costs. The analysis further reveals that TCM treatment cost exhibits the highest correlation with the total cost (0.735), which is consistent with its 41% share of total costs. This strong association can be attributed to the diversity of TCM treatments (such as acupuncture, tuina, and physiotherapy), which play a crucial role in stroke rehabilitation by requiring long and frequent treatment cycles, thereby driving up overall costs.^[14]^ The results of the gray correlation of stroke hospitalization costs are shown in Table 2.

**Table 2:** Gray correlation of stroke hospitalization costs.

Cost category	Relatedness	Arrange in order
TCM treatment fee	0.735	1
Diagnostic fee	0.666	2
Western drug costs	0.651	3
General medical services	0.629	4
Cost of proprietary Chinese medicines	0.628	5
Chinese herbal medicine costs	0.627	6
Material cost	0.622	7
Nursing costs	0.621	8

Source: Primary data collection.

TCM = traditional Chinese medicine.

### 4.2. Analysis of factors influencing hospitalization costs for stroke patients

#### 4.2.1. Univariate analysis of the impact of hospitalization costs in stroke patients

The Mann–Whitney *U* rank sum test and the Kruskal–Wallis *H* rank sum test indicated that age had a statistically significant effect on both the number of hospitalization days (*P* < .05) and treatment costs (*P* < .05). Specifically, as age increased, the number of hospitalization days tended to decrease, while treatment costs tended to increase. Table 3 presents the univariate analysis results of hospitalization costs for stroke patients.^[15]^ This indicates that the medical burden of stroke patients is mainly related to age: elderly patients (aged ≥80 years) and young patients (aged 20–39 years) incur higher costs, while the group aged 60 to 79 years is relatively more economical. Gender has almost no impact on the length of hospital stay and medical expenses. The results of the univariate analysis of hospitalization costs for stroke patients are shown in Table 3.

**Table 3:** Univariate analysis of the impact of hospitalization costs for stroke patients.

Hallmark	Number of cases/percentage	Average number	Days of hospitalization, *M* (P25–P75)	*P*	Average number	Cost of treatment ($), *M* (P25–P75)	*P*
Distinguishing between the sexes				.557			.967
A male	484 (66.67)	13.08	14 (10–16)		1144.81	2283.68 (10,903.39–21,479.58)	
Females	242 (33.33)	13.48	14 (10–16)		2372.79	2227.33 (11,399.12–20,658.63)	
Age (yr)				.000**			.000**
20–39	7 (0.96)	13.86	15 (12–16)		2671.70	2647.19 (14,165.22–20,909.790)	
40–59	131 (18.04)	10.44	14 (9.25–16)		2501.68	2622.31 (11,570.79–22,239.89)	
60–79	448 (61.71)	6.95	13 (9–16)		1128.26	2051.00 (10,054.21–19,946.48)	
≥80	140 (19.28)	8.41	15 (12–17)		2612.05	2558.27 (12,940.86–22,521.72)	

Source: Primary data collection. **Statistical significance, *P* < .01. In this specific context, together with the *P*-value of .000, it strongly indicates that "age" is a very important factor with high statistical significance affecting the length of hospital stay or treatment cost of patients.

#### 4.2.2. Multiple linear regression analysis

In this study, variables that reached statistical significance (*P* < .05) in the 1-way analysis of variance were incorporated into a multiple linear regression model. The significance level was set at α = 0.05. The final model yielded *R*^2^ = 0.553 and *F* = 297.953 (*P* < .001), and all variance inflation factors (VIFs) were below 10, indicating that multicollinearity was not a concern and that the model was well constructed. Controlling for covariates, the results revealed that the length of hospital stay had a statistically significant effect on hospitalization costs for stroke patients (*P* < .05); specifically, an increase in the number of hospitalization days was associated with a significant rise in total costs.^[15]^ In contrast, the effect of age on hospitalization costs was not statistically significant (*P* > .05), That is, the hospitalization expenses of stroke patients are only strongly correlated with the length of hospital stay, and have no correlation with age or gender. These findings suggest that additional factors, beyond those included in the current model, may also significantly influence hospitalization costs and warrant further investigation. The results of the multiple linear regression analysis of the hospitalization cost of stroke patients are shown in Table 4.

**Table 4:** Multiple linear regression analysis of hospitalization costs for stroke patients.

Variant	Standardized coefficient, beta	*t*	*P*
A constant (math.)	–	1.775	.076
Sex (control = male)	0.005	0.186	.852
(A person’s) age	−0.031	−1.223	.222
Length of hospitalization	0.746	29.872	.000**

Source: Primary data collection.

## 5. Analysis of factors influencing the cost of hospitalization for “facial palsy”

### 5.1. Cost composition of disease types

#### 5.1.1. Information on hospitalization costs

From January 1, 2023 to December 31, 2023, a total of 1 year, 294 patients with facial palsy were discharged from the sample hospital. The average total hospitalization cost of 294 patients was 1457.65 dollars, the maximum value of the cost was 3228.54 dollars, and the minimum value was 363.16 dollars. Among them, the average cost of general medical service was 78.59 dollars, the minimum value was 13.24 dollars, the maximum value was 438.18 dollars, and the average proportion of general medical service cost was 5.39%; the average cost of nursing care was 21.01 dollars, the minimum value was 4.46 dollars, the maximum value was 43.48 dollars, and the average proportion of nursing care cost was 1.44%; the average cost of diagnosis was 222.33 dollars, the minimum value was 0.42 dollars, the maximum value was 739.71 dollars, and the average diagnostic fee is 15.25%; TCM treatment fee is 700.39 dollars on average, the minimum value is 17.42 dollars, the maximum value is 2076.32 dollars, and the average TCM treatment fee is 48.05%; Western medicine fee is 150.11 dollars on average, the minimum value is 0 dollars, the maximum value is 648.40 dollars, and the average Western medicine fee is 10.3%. The average cost of proprietary Chinese medicines was 12.36 dollars, the minimum value was 0 dollars, the maximum value was 188.69 dollars, and the average proportion of the cost of proprietary Chinese medicines was 0.85%; the average cost of herbal medicines was 89.44 dollars, the minimum value was 0 dollars, the maximum value was 358.62 dollars, and the average proportion of the cost of herbal medicines was 3.45%; the average cost of materials was 9.11 dollars, the minimum value was 0 dollars, the maximum value was 192.47 dollars, and the average proportion of the cost of materials was 0.00%. The components of hospitalization costs for facial paralysis are shown in Table 5.

**Table 5:** Analysis of cost components of facial palsy hospitalization (unit: dollars).

Sports event	Minimum value	Average value	Maximum values
Total cost of hospitalization	363.16	1457.65	3228.54
General medical services	13.24	78.59	438.18
Nursing costs	4.46	21.01	43.48
Diagnostic fee	0.42	222.33	739.71
TCM treatment fee	17.42	700.39	2076.32
Western drug costs	0	1077.04	648.40
Cost of proprietary Chinese medicines	0	12.36	188.69
Chinese herbal medicine costs	0	89.44	358.62
Material cost	0	9.11	192.47

Source: Primary data collection.

TCM = traditional Chinese medicine.

#### 5.1.2. Gray correlation analysis of hospitalization costs

Using the average total hospitalization cost as the reference, we calculated the absolute differences between each cost component for facial palsy and the average cost. Based on the maximum and minimum differences, correlation coefficients were determined for each cost category. In descending order, the correlation coefficients were: TCM treatment fee, diagnostic fee, Western drug costs, Chinese herbal medicine costs, general medical services, nursing costs, cost of proprietary Chinese medicines, and material cost. Notably, this ranking is consistent with that obtained from the percentage composition of hospitalization costs.

The study found that TCM treatment costs were strongly correlated with total hospitalization costs, with a correlation coefficient of 0.992, substantially higher than the diagnostic cost of 0.10. The clinical treatment for facial paralysis primarily relies on TCM acupuncture and Chinese herbal medicine, with Western medicines used as supplements.^[16]^ Due to the multiple treatment steps and extended treatment cycles inherent in TCM, its cost share is high, thereby contributing to this strong association. The results of the gray correlation of hospitalization costs for facial paralysis are shown in Table 6.

**Table 6:** Gray correlation of facial palsy hospitalization costs.

Cost category	Relatedness	Arrange in order
TCM treatment fee	0.992	1
Diagnostic fee	0.714	2
Western drug costs	0.675	3
Chinese herbal medicine costs	0.648	4
General medical services	0.641	5
Nursing costs	0.621	6
Cost of proprietary Chinese medicines	0.618	7
Material cost	0.617	8

Source: Primary data collection.

TCM = traditional Chinese medicine.

### 5.2. Analysis of factors influencing hospitalization costs for patients with facial palsy

#### 5.2.1. A unifactorial analysis of the impact of hospitalization costs in patients with facial palsy

The Mann–Whitney *U* rank sum test and the Kruskal–Wallis *H* rank sum test indicated a statistically significant difference in treatment costs by gender (*P* < .05). Specifically, male patients incurred higher treatment costs, while female patients incurred lower costs.^[15]^ That is, the treatment cost of facial paralysis is mainly related to gender (lower for females) and has no correlation with age or length of hospital stay. The results of univariate analysis of hospitalization cost of facial paralysis are shown in Table 7.

**Table 7:** Univariate analysis of the influence of hospitalization costs for patients with facial palsy.

Hallmark	Number of cases/percentage	Average number	Days of hospitalization, *M* (P25–P75)	*P*	Average number	Cost of treatment ($), *M* (P25~P75)	*P*
Distinguishing between the sexes				.095			.039*
A male	162 (55.1)	12.51	14 (10–15)		1144.81	1734.60 (9795.63–12,446.08)	
Females	132 (44.9)	13.26	14 (11–15)		2372.79	1524.05 (7726.33–13,747.17)	
Age (yr)				.127			.057
<20	3 (1.02)	17	15 (15–21)		1332.30	1793.39 (7934.22–1654.26)	
20–39	63 (21.43)	12.17	13 (10–15)		1292.79	1224.17 (6865.65–11,626.46)	
40–59	111 (37.76)	12.85	14 (10–15)		1465.40	1867.63 (9949.92–402.4)	
60–79	108 (36.73)	13.06	14 (11–15)		1530.41	1529.75 (7530.76–13,542.12)	
≥80	9 (3.06)	13.56	13 (11–17)		1684.92	1728.34 (9414.59–13,977.21)	

Source: Primary data collection. *Significance marker that follows the *P*-value of .039, clearly indicating that gender is a significant factor affecting the hospitalization costs of patients with facial paralysis.

#### 5.2.2. Multiple linear regression analysis

In this study, variables that were statistically significant (*P* < .05) in the univariate analysis were incorporated into a multiple linear regression model. The significance level was set at α = 0.05, and the final model yielded *R*^2^ = 0.512, *F* = 101.425, *P* < .001, indicating statistical significance. All VIFs were below 10, confirming the absence of multicollinearity and ensuring the model’s validity.

Controlling for covariates, the results revealed that the length of hospital stay had a statistically significant effect on hospitalization costs for facial palsy (*P* < .05). This suggests that reducing hospital stay duration could be a key strategy for lowering healthcare costs.^[15]^ In contrast, gender and age did not show statistically significant effects (*P* > .05). Although age was significant in univariate analysis, its impact disappeared when other variables were accounted for in the multivariate model. This finding implies that hospitalization costs for facial palsy are primarily driven by LOS, while gender and age may influence costs through indirect pathways. The study also suggests that additional factors affecting hospitalization costs remain unidentified and warrant further investigation. The treatment cost is entirely dominated by the length of hospital stay (the longer the hospitalization, the higher the cost) and has no correlation with the patient’s gender or age. The results of the multiple linear regression of hospitalization costs for patients with facial palsy are shown in Table 8.

**Table 8:** Multiple linear regression analysis of hospitalization costs for facial palsy patients.

variant	Standardized coefficient, beta	*t*	*P*
A constant (math.)	–	−0.897	.371
Sex (control = male)	0.046	1.081	.281
(A person’s) age	0.069	1.618	.107
Length of hospitalization	0.697	16.839	.000**

Source: Primary data collection. **Statistical significance. In this specific context, together with an extremely small *P*-value of .000, it clearly indicates that “length of hospitalization” is the most critical and significant factor determining the hospitalization costs of patients with facial paralysis.

## 6. Analysis of factors influencing the cost of hospitalization for “bone paralysis”

### 6.1. Composition of the cost of a disease

#### 6.1.1. Information on hospitalization costs

From January 1, 2023 to December 31, 2023, a total of 1 year, 244 cases of bone paralysis patients were discharged from the sample hospital. The average total hospitalization cost of the 244 patients was 1406.88 dollars, with the maximum value of the cost being 5489.53 dollars, and the minimum value of the cost being 481.44 dollars. Among them, the average cost of general medical service was 75.93 dollars, the minimum value was 18.54 dollars, the maximum value was 224.94 dollars, and the average proportion of general medical service cost was 5.4%; the average cost of nursing care was 19.66 dollars, the minimum value was 12.26 dollars, the maximum value was 45.16 dollars, and the average proportion of nursing care cost was 1.4%. The average cost of diagnosis was 311.48 dollars, the minimum value was 7.67 dollars, the maximum value was 1103.65 dollars, and the average proportion of diagnosis cost was 22.14%; the average cost of Chinese medicine treatment was 469.93 dollars, the minimum value was 0 dollars, the maximum value was 1392.57 dollars, and the average proportion of Chinese medicine treatment cost was 33.4%; the average cost of Western medicine was 237.33 dollars, the minimum value was 0 dollars, the maximum value was 1307.92 dollars, and the average proportion of Western medicine cost was 16.87%; the average cost of proprietary Chinese medicine was 34.68 dollars, the minimum value is 0 dollars, the maximum value is 228.54 dollars, and the cost of proprietary Chinese medicines accounted for an average of 2.47%; Chinese herbal medicines cost an average of 45.24 dollars, the minimum value is 0 dollars, the maximum value is 224.60 dollars, and the cost of Chinese herbal medicines accounted for an average of 3.22%. The average cost of materials was 212.64 dollars, the minimum value was 0 dollars, the maximum value was 4130.71 dollars, and the average cost of materials was 15.11%. The composition of hospitalization cost of bone paralysis is shown in Table 9.

**Table 9:** Analysis of bone paralysis hospitalization cost components (unit: dollars).

Sports event	Minimum value	Average value	Maximum values
Total cost of hospitalization	481.44	1406.88	5489.53
General medical services	18.54	75.93	224.94
Nursing costs	12.26	19.66	45.16
Diagnostic fee	7.67	311.48	1103.65
TCM treatment fee	0	469.93	1392.57
Western drug costs	0	237.33	1307.92
Cost of proprietary Chinese medicines	0	34.68	228.54
Chinese herbal medicine costs	0	45.24	224.60
Material cost	0	212.64	4130.71

Source: Primary data collection.

TCM = traditional Chinese medicine.

#### 6.1.2. Gray correlation analysis of hospitalization costs

Using the average total hospitalization cost as the reference, we calculated the absolute differences between each individual cost component for bone paralysis and the average total cost. The maximum and minimum differences were then used to determine the correlation coefficients for each cost category. Table 10 presents these results.

**Table 10:** Gray correlation of hospitalization costs for bone paralysis.

Cost category	Relatedness	Arrange in order
TCM treatment fee	0.946	1
Diagnostic fee	0.879	2
Western drug costs	0.853	3
Material cost	0.837	4
General medical services	0.8	5
Chinese herbal medicine costs	0.791	6
Cost of proprietary Chinese medicines	0.788	7
Nursing costs	0.783	8

Source: Primary data collection.

TCM = traditional Chinese medicine.

In descending order, the correlation coefficients with total hospitalization cost were: TCM treatment fee, diagnostic fee, Western drug costs, material cost, general medical services, Chinese herbal medicine costs, cost of proprietary Chinese medicines, and nursing costs. Notably, the ranking obtained from gray correlation analysis aligns closely with the percentage composition of hospitalization costs.

Gray correlation analysis revealed that TCM treatment costs (correlation coefficient: 0.946) and diagnostic costs (0.879) had the strongest associations with total hospitalization costs. In clinical practice, diagnosis often depends on specialized tests, which are expensive, while TCM treatments involve a diverse range of therapies over extended durations, contributing significantly to total costs. These findings provide a solid foundation for further medical cost research.^[17]^ The results of the gray correlation of hospitalization costs for bone paralysis are shown in Table 10.

### 6.2. Analysis of factors influencing hospitalization costs for patients with bone paralysis

#### 6.2.1. A unifactorial analysis of the impact of hospitalization costs in patients with bone paralysis

The Mann–Whitney *U* rank sum test and the Kruskal–Wallis *H* rank sum test indicated a statistically significant relationship between age and hospitalization duration (*P* < .05) and between gender, age, and treatment costs (*P* < .05). Older patients had longer hospital stays and higher treatment costs, while male patients incurred lower treatment costs than female patients.^[15]^ This indicates that patients’ medical burden (expenses) is affected by gender, with male patients incurring higher costs. Both the length of hospital stay and medical expenses increase with age, meaning elderly patients face the greatest pressure. Gender does not prolong the hospital stay, but age is a key driving factor. This reminds the medical system to focus on optimizing nursing plans for the elderly population and controlling differences in medical expenses. The results of the univariate analysis of the hospitalization cost of bone paralysis patients are shown in Table 11.

**Table 11:** Univariate analysis of the impact of hospitalization costs for patients with bone paralysis.

Hallmark	Number of cases/percentage	Average number	Days of hospitalization, *M* (P25–P75)	*P*		Average number	Cost of treatment ($), *M* (P25–P75)	*P*
Distinguishing between the sexes					.171			.039*
A male	79 (32.38)	11.3	10 (9–14)			1675.87	1093.75 (5961.15–10,417.72)	
Females	165 (67.62)	11.83	11 (9–13.5)			1656.31	1274.01 (6615.81–11,296)	
Age (yr)				.002**				.004**
<20	2 (0.82)	12	12 (8–16)			1060.15	1078.56 (5596.66–2142.26)	
20–39	19 (7.79)	10.05	10 (7–11)			1472.40	851.41 (5450.58–9827.18)	
40–59	66 (27.05)	10.77	10 (8–13)			1566.87	1132.82 (5849.74–10,074.04)	
60–79	129 (52.87)	11.98	11 (10–14)			1670.93	1246.45 (7085.42–11,104.52)	
≥80	28 (11.48)	13.36	12 (10–15.75)			2022.24	1523.73 (7132.55–15,649.38)	

Source: Primary data collection. *Gender has no significant effect on the length of hospital stay, but a significant effect on treatment costs. **Age has a highly significant effect on both the length of hospital stay and treatment costs, and is the most influential factor.

#### 6.2.2. Multiple linear regression analysis

This study incorporated variables that were statistically significant (*P* < .05) in the univariate analysis into a multiple linear regression model. The significance level was set at α = 0.05, and the final model yielded *R*^2^ = 0.288, *F* = 32.431, *P* < .001, indicating statistical significance. All VIFs were below 10, confirming the absence of multicollinearity and ensuring the model’s validity.

Controlling for covariates, the results showed that length of hospital stay had a statistically significant impact on hospitalization costs for bone paralysis (*P* < .05). Specifically, longer hospital stays were associated with higher total hospitalization costs.^[15]^ However, gender and age did not have statistically significant effects on hospitalization costs (*P* > .05). That is, the sole core driving factor of treatment costs is the length of hospital stay (the longer the hospitalization, the higher the cost), while factors such as the patient’s gender, age, and basic expenses have no substantial impact. These findings suggest that additional factors influencing hospitalization costs remain unidentified and warrant further investigation. The results of multivariate linear regression of hospitalization cost of bone paralysis patients are shown in Table 12.

**Table 12:** Multiple linear regression analysis of hospitalization costs for bone palsy patients.

Variant	Standardized coefficient, beta	*t*	*P*
A constant (math.)	–	−1.174	.242
Sex (control = male)	0.017	0.309	.757
(A person’s) age	0.046	0.820	.413
Length of hospitalization	0.548	9.913	.000**

Source: Primary data collection.

## 7. Results

### 7.1. Strokes

This study analyzes the structure of hospitalization costs and the factors influencing stroke-related expenses. The results indicate that Chinese medicine treatment accounted for the largest proportion (41%) of total costs. This is primarily due to acupuncture treatments targeting acupoints such as Baihui and Fengchi, which require multiple sessions; the use of Chinese medicinal preparations, such as Tonifying Yang Huiwu Tang, prescribed based on dialectical treatment principles; and extended rehabilitation training in TCM techniques, all of which contribute to higher costs.^[14]^

Diagnostic costs made up 16.2% of total expenses, primarily due to the reliance on costly imaging tests (e.g., cranial computed tomography/magnetic resonance imaging [MRI]), laboratory analysis of various blood markers, and specialized assessments of neurological function and daily living ability, all of which contribute to higher costs.^[18]^

The analysis of cost-related factors revealed that length of hospital stay was the only significant determinant (Beta = 0.746), directly impacting total expenses. While age showed a correlation in univariate analysis, its effect was not significant in the multivariate model. To enhance cost model accuracy, expenses related to common stroke complications, such as lung infections and cardiovascular diseases, should be incorporated.

### 7.2. Facial paralysis

Research indicates that Chinese medicine accounts for 48.05% of hospitalization costs for facial paralysis, underscoring the prominence of traditional therapies such as acupuncture and tuina. Acupuncture and moxibustion stimulate acupoints like Zanzhu to regulate facial qi and blood circulation, promoting nerve recovery. These treatments require multiple sessions over several weeks, leading to higher costs. Additionally, tui-na enhances muscle tension based on the treatment regimen, further contributing to expenses.^[19]^

Diagnostic costs comprised 15.25% of total expenses, primarily due to tests for ruling out central facial paralysis. These include high-cost imaging techniques like MRI/computed tomography for detecting brain lesions, electromyography for assessing facial nerve electrophysiology, and TCM diagnostic methods for symptom identification, all of which contribute to overall costs.^[18]^

The primary factor driving increased hospitalization costs is the LOS. Longer hospital stays directly raise total expenses. Additionally, male patients tend to have higher hospitalization costs than female patients, potentially due to poorer lifestyle habits that complicate treatment, lower adherence to medical recommendations leading to prolonged treatment cycles, and greater muscular complexity, which may necessitate more intensive nerve repair interventions.

### 7.3. Bone paralysis

In the study of bone paralysis hospitalization costs, TCM treatments accounted for 33.4% of total expenses, highlighting their widespread application. Acupuncture and moxibustion, targeting acupoints such as Ren Yu and Calyx, require multiple sessions, leading to increased costs. Additionally, Chinese medicine transdermal therapy, which utilizes low- and medium-frequency electrotherapy equipment, incurs rising costs due to prolonged equipment use and consumables. Classical prescriptions, such as tonic Chinese medicine and Gui Zhi Tang, formulated based on evidence-based treatment principles, contribute to costs from procurement to administration.^[20]^

Diagnostic costs comprised 22.14% of total expenses, primarily due to reliance on X-rays and MRI for joint degeneration assessment, both of which involve high equipment costs. Additional expenses arose from inflammatory marker tests and diagnostic evaluations conducted by Chinese medicine practitioners.^[18]^

Length of hospital stay was the only significant factor influencing total costs, with longer stays leading to higher expenses. Women had higher hospitalization costs, potentially due to a greater preference for integrated TCM treatments, which involve longer and more adherent treatment regimens. Additionally, physiological and physical differences may necessitate frequent adjustments in treatment plans, further increasing costs.^[15]^

## 8. Discussion

### 8.1. Optimizing health insurance payment mechanisms

Reforming the health insurance payment mechanism is an urgent priority. Under the current DRG payment system, cost weights for TCM treatment items should be adjusted to reflect their unique therapeutic contributions.^[21]^ TCM treatments, including acupuncture, moxibustion, transdermal herbal therapy, and classical formula applications, play a crucial role in treating bone paralysis. Properly calibrating these cost weights would better capture the intrinsic value of TCM techniques, discourage unnecessary medical interventions, and establish a more balanced compensation system. This reform seeks to allocate health insurance resources more effectively toward TCM treatments while incentivizing medical institutions to expand their specialized Chinese medicine services.

### 8.2. Enhanced refinement of inpatient management

Implementing refined hospitalization management is crucial for cost control. First, it minimizes nonessential hospital stays and reduces unnecessary resource consumption. For instance, optimizing diagnostic and treatment processes enhances efficiency, preventing prolonged hospitalizations caused by avoidable delays. Second, promoting alternative treatment models, such as outpatient rehabilitation for bone paralysis, can further reduce costs. Many patients can transition to outpatient rehabilitation once their condition stabilizes, alleviating the financial burden of hospitalization while lowering overall healthcare expenses.

### 8.3. Building a multidimensional research network

Developing a multidimensional research network is essential for thoroughly investigating the cost-effectiveness of bone paralysis treatment. Machine learning algorithms can facilitate in-depth mining of extensive medical data, while multicenter follow-up studies can enhance the representativeness of clinical information. Additionally, cost-effectiveness analysis allows for a systematic evaluation of the clinical benefits and economic feasibility of high-cost TCM treatments. Future research should incorporate outcome indicators such as complication rates and neurological recovery metrics to establish a cost-effectiveness model. This will provide robust evidence for optimizing resource allocation between TCM and Western medicine, ensuring a more rational and efficient use of healthcare resources.^[22]^

## Author contributions

**Conceptualization:** Chenhao Ma, Xiping Li.

**Data curation:** Chenhao Ma.

**Formal analysis:** Chenhao Ma, Xiping Li.

**Funding acquisition:** Xiping Li.

**Investigation:** Chenhao Ma, Tong Qin, Songkun Ding, Wei Hu, Tao He.

**Methodology:** Songkun Ding.

**Project administration:** Xiping Li.

**Resources:** Xiping Li.

**Software:** Chenhao Ma.

**Supervision:** Xiping Li, Tong Qin, Wei Hu, Tao He.

**Validation:** Chenhao Ma.

**Visualization:** Chenhao Ma.

**Writing – original draft:** Chenhao Ma.

**Writing – review & editing:** Chenhao Ma, Xiping Li.
